# Effects of migration on tuberculosis epidemiological indicators in low and medium tuberculosis incidence countries: A systematic review

**DOI:** 10.1016/j.jctube.2021.100225

**Published:** 2021-02-22

**Authors:** Sarah Jackson, Zubair Kabir, Catherine Comiskey

**Affiliations:** a School of Nursing and Midwifery, Trinity College Dublin, University of Ireland, Ireland; b School of Public Health, University College Cork, Ireland

**Keywords:** Epidemiology, TB, Meta-analysis, Migrant, Immigration, Immigrant

## Abstract

**Background:**

Tuberculosis (TB) remains one of the top ten causes of death each year globally. While the risk of migrant TB is linked to the TB incidence in their country of origin, the migration process can increase the TB risk.

**Objective:**

We aimed to synthesis the evidence on key differences in the epidemiological profile of TB between migrants from high TB incidence birth countries and non-migrants resident in low to medium incidence TB countries.

**Methods:**

We conducted a systematic review where the population was all active TB cases in countries with low to medium TB incidence (<40/100,000 population), the exposure was migration to a low or medium TB incidence country and the comparator was non-migrant TB cases in low or medium incidence countries. Overall proportions were compared between migrants and non-migrants, using Fisher’s exact test. Meta-analysis of proportions was carried out for the primary outcome (active TB) while meta-analyses of odds ratios (ORs) were performed using a random effects model for secondary outcomes; sputum-smear positivity, any first line drug resistance, multi-drug resistance (MDR), clustered cases, HIV coinfections and successful treatment. Heterogeneity was evaluated and sources were investigated using subgroup and sensitivity analysis.

**Results:**

Significant differences were found in the overall proportions of high TB incidence migrants and non-migrants for MDR cases, clustered cases, HIV coinfections and successful treatment, as well as a significant difference in the OR among MDR cases (3.91).

**Conclusion:**

This review has demonstrated significant differences in key epidemiological indicators between high TB incidence migrants and non-migrants, indicating policy implications.

## Highlights

• Differences found in epidemiology of high TB incidence migrants and non-migrants.
• Differing odd ratios found among migrants for MDR (3.91).
• Differing migrant proportions in HIV co-infections, MDR, clusters, treatment success.
• Gap in the literature found regarding reporting specific data on migrant TB origin.
• Further research required to address this gap and inform TB elimination programmes.

## 1 Introduction:

Tuberculosis (TB) continues to kill more people annually than any other infectious disease and is one of the top ten causes of death each year globally. The World Health Organisation (WHO) estimated that 1.5 million people died from TB during 2019 alone.[1] While the risk of TB infection and disease in migrants is linked to the level of TB incidence in their country of origin, the process of migration itself can increase the risk of being infected or developing TB disease.[2] This can occur due to increased risk of exposure along migration routes as well as the various social and behavioural determinants on arrival in the host country.[3], [4], [5]

Much has been written on the topic of migrant TB, particularly in Europe, but most of these studies have treated migrants as a homogenous group with broad definitions such as those who were born outside or holding nationality different to that of the country under study.[6], [7], [8] As noted by Hanway et al, the category of “foreign-born” essentially refers to being born in any one of 197 potential other countries.[9] Migrants are a highly diverse group in most countries, manifesting through their country of origin, mode of migration, socioeconomic grouping, demographic profile and health status. This leads to differing chances of importing existing infection, acquiring infection once arrived and accessing health services if they develop TB. A large study found that migrants from other European Union (EU) countries did not contribute significantly to the overall incidence of TB within the EU. Instead, most of the migrant cases were found to be from high TB incidence countries outside the EU [7].

Although narrative reviews of quantitative data and systematic reviews of qualitative data on the topic exist, no systematic review has been performed on the quantitative data.[3], [10] As it is neither possible nor desirable to eliminate human migration, a better understanding of the effects of migration on the epidemiology of TB is needed in order to continue working towards the global goal of TB elimination.[11]

This systematic review aims to investigate how migration from high TB incidence countries affects the epidemiology of TB in low to medium incidence countries by comparing key epidemiological outcomes between active TB cases diagnosed in migrants from high TB endemicity birth countries and non-migrant cases reported in a low or medium incidence country. The review outcomes investigated were selected as indicators of potential transmission and case complexity based on clinical evidence.

## 2 Methods

The review adheres to the Preferred Reporting Items for Systematic Reviews and Meta-Analysis (PRISMA) and Meta-analysis of Observational Studies in Epidemiology (MOOSE) guidelines. [12], [13] The protocol was prospectively registered on the International Prospective Register of Systematic Reviews (PROSPERO) as CRD42018095038.[14]

### 2.1 PECO

The review question utilised a PECO model where the population was all active TB cases diagnosed and resident in countries with low to medium TB incidence (<40/100,000 population).[15] The exposure group was TB cases from high TB incidence (≥40/100,000 population) birth countries who migrated to a low or medium TB incidence country of residence. The comparator group was TB cases born in the low or medium TB incidence country of the study. Migrant status was assigned based on country of birth being different to the country of the study.

### 2.2 Outcomes

The primary outcomes extracted were the number and proportion of active TB cases in the exposure and comparator categories. Six secondary outcomes were also assessed for the exposure and comparator categories: sputum smear positive cases; cases resistant to any first line anti-TB drug; multi-drug resistant (MDR) cases; cases clustered by genotyping or whole genome sequencing (WGS); HIV coinfected cases; and successfully treated cases.[15].

### 2.3 Inclusion criteria

Observational studies (including cohort, case control and cross-sectional studies) and publications reporting on routinely collected health data (RCD) using prospective, retrospective or cross-sectional designs were included. A start date of 2010 was selected in order to avoid cross over periods where the same country was categorised as a high TB incidence country in earlier publications and a low or medium TB incidence in later publications. Animal studies, cases diagnosed/ resident in countries with high TB incidence, latent TB, internal migration within countries, studies where country of birth was missing for >5% of foreign-born TB cases, case studies, case series, studies published prior to 2010 and non-English language abstracts were excluded. Full details of inclusion and exclusion criteria are detailed in the protocol and Table S1.[14]

### 2.4 Data sources

Five electronic databases; Medline (EBSCO), EMBASE (EMBASE), CINAHL (EBSCO), Scopus (Elsevier) and ScieLo (Web of Science) were searched using controlled vocabulary and key words. Websites of the WHO, European Centre for Disease Prevention and Control (ECDC), Health Protection Scotland, Public Health Agency, Northern Ireland, Public Health England, Department of Health Australia, Public Health Surveillance, New Zealand, Public Health Agency Canada and Centers for Disease Control, United States and OpenGrey (Grey Net) were searched by key words. The initial search was performed in August 2019 and updated in March 2020.

### 2.5 Search terms

The search strategy utilised three concepts; TB, migrants and active or confirmed status. Key words for the tuberculosis concept included *Tuberculosis OR Tuberculoses OR Tuberculous OR Tuberculoid OR “Koch’s Disease” OR “Kochs Disease” OR “Koch Disease” OR “potts disease” OR “pott’s disease” OR “pott disease” OR scrofula OR phthisis*. Key words for the migrant concept included transient* OR migrant* OR emigrant* OR immigrant* OR refugee* OR “asylum seeker*” OR emigration OR immigration OR relocation OR relocate*. Keywords for the final concept included active OR confirm* OR positiv*. Table S2 details the search terms as used in EMBASE.

### 2.6 Study selection and review

Articles identified by the search strategy were independently evaluated by two reviewers at each stage of the review (title and abstract screening, full text review, data extraction and critical appraisal) using Covidence™ software. Variables extracted are detailed in Table S3. The National Institute for Health (NIH) quality appraisal tool for cohort and cross sectional studies was used (Table S4).[16] As the exposure was country of birth, multiple assessment of exposure was not evaluated. Bibliographies of included studies were hand searched.

### 2.7 Statistical analysis

Overall proportions were compared across exposures using Fisher’s exact test. Meta-analysis of primary outcome proportions was performed using the Metaprop command which produces weighted sub-group and pooled estimates with inverse-variance weights obtained from a random-effects model in Stata 16™ (Stata Statistical Software: Release 16. College Station: StataCorp LP).[17], [18] Odds ratios for secondary outcomes were calculated using an inverse variance statistical model with a random effects analysis model in Review Manager 5.3. Meta-analyses results were displayed via forest plots allowing use of visual inspection, Cochran’s Q-tests and I^2^ to evaluate heterogeneity.[19] Studies whose primary outcome was defined as a secondary outcome of this review were excluded from the meta-analysis for that outcome as the numerator was the same as the denominator.

### 2.8 Subgroup and sensitivity analysis

Due to the clinical heterogeneity observed among included studies, they were further grouped according to clinical categories for sensitivity analysis; all diagnostic types, drug resistant TB, extrapulmonary only TB, pulmonary TB, clustered cases, TB in pregnancy, paediatric cases and deceased donors/ donor recipients. Methodological heterogeneity was explored via the per protocol subgroups; year of publication, study design, study setting, geographical region and TB incidence level of study country. Outcomes were analysed by the total studies that reported the outcome, the clinical categories and per protocol subgroups.

## 3 Results

**Fig. 1:**
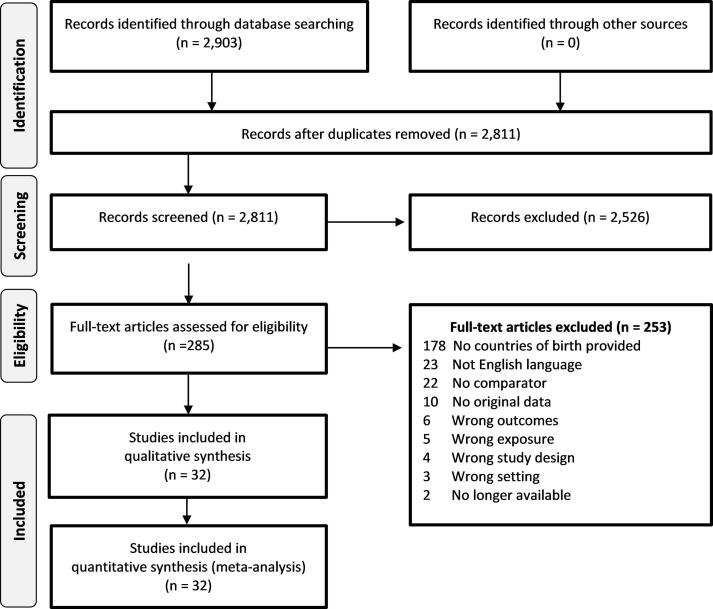
PRISMA flow diagram of retrieved studies. *No comparator = migrant only study population; no original data = systematic/ narrative reviews or editorials; wrong outcomes = outcomes other than active TB; wrong exposure = where migrant study definition did not match migrant review definition, wrong setting = high TB incidence country.

Thirty-two studies identified by the search and screening process (Fig. 1) met the inclusion criteria requirements, comprising a total of 93,235 TB cases (median sample size = 98.5; range: 6–73,945). Sixteen included studies were conducted in Europe, 12 were set in Middle Eastern / Western Asian countries, two were from Australia and one each were from Guadaloupe and the United States. Twenty-five studies were set in low TB incidence countries while seven were in medium TB incidence countries. The majority of studies were cohort studies, 23 of which were retrospective and one was prospective. The remaining eight included studies were cross sectional design. The most common study setting was hospitals (n = 16), followed by studies that utilised routinely collected health data (n = 10), such as surveillance or programmatic data. Table 1 outlines the main characteristics of included studies.

**Table 1:** Characteristics of included studies.

**Author**	**Country**	**TB incidence**	**Study design**	**Study setting**	**Study outcome**	**Study size**	**Year of study**	**Study duration months**	**Study follow up months**	Domains with low RoB*
Aguayo 2010 [20]	Spain	Low	Cross sectional	Hospital	Extra-pulmonary TB	20	1999–2009	132	not reported	n/a
Al-Hajoj 2015 [21]	Saudi Arabia	Low	Cross sectional	Routinely collected health data	Extra-pulmonary TB - culture confirmed	381	2009–2010	12	not reported	6/13
Azarkar 2016 [22]	Iran	Medium	Retrospective cohort	TB clinic	Pulmonary TB	85	2010–2011	24	not reported	8/13
Bartu 2010 [23]	Czech Republic	Low	Retrospective cohort	Hospital	MDR-TB	50	2001–2009	108	108	10/13
Bendayan 2011 [24]	Israel	Low	Retrospective cohort	Hospital	MDR-TB - hospitalised new	132	2000–2005	60	24	9/13
Bishara 2015 [25]	Israel	Low	Retrospective cohort	Routinely collected health data	Active TB in pregnancy	6	2002–2012	132	not reported	9/13
Broderick 2018 [26]	United Kingdom	Low	Retrospective cohort	Hospital	Extra-pulmonary TB - bone / joint	29	2012–2014	24	not reported	9/13
Coll 2013 [27]	Spain	Low	Retrospective cohort	Hospital	Active TB in donors and donor recipients	6	1998–2011	162	162	9/13
Cruz-Ferro 2014 [28]	Spain	Low	Retrospective cohort	TB clinic	Active TB	12,615	1996–2011	192	192	8/13
Doĝru 2017 [29]	Turkey	Medium	Retrospective cohort	Hospital	Pulmonary TB - new	211	2010–2013	48	not reported	10/13
Fallico 2014 [30]	Italy	Low	Retrospective cohort	Hospital	Active TB	339	2006–2009	48	not reported	9/13
Ferdinand 2013 [31]	Guadeloupe	Low	Prospective cohort	Community setting	Active TB - culture confirmed	129	1999–2005	81	not reported	10/13
Goblirsch 2014 [32]	Saudi Arabia	Low	Retrospective cohort	Hospital	Extra-pulmonary TB & HIV coinfection	39	2008–2012	39	not reported	9/13
Helbling 2014 [33]	Switzerland	Low	Retrospective cohort	Routinely collected health data	MDR-TB	51	2003–2010	115	24	10/13
Jagielski 2010 [34]	Poland	Medium	Retrospective cohort	TB clinic	MDR-TB	117	2004	12	12	10/13
Jawad 2014 [35]	Bahrain	Medium	Retrospective cohort	Routinely collected health data	Active TB	1,584	2000–2006	84	Not reported	9/13
Jensenius 2016 [36]	Norway	Low	Retrospective cohort	Routinely collected health data	Drug resistant TB	88	1995–2014	240	not reported	10/13
Jones 2017 [37]	Australia	Low	Retrospective cohort	Routinely collected health data	Active TB	171	2006–2015	108	Not reported	n/a
Kentley 2017 [38]	United Kingdom	Low	Retrospective cohort	Hospital	Extra-pulmonary intestinal TB	61	2008–2014	84	not reported	8/13
Krogh 2010 [39]	Norway	Low	Retrospective cohort	Hospital	Paediatric TB	24	1998–2009	124	124	10/13
Lumb 2013 [40]	Australia	Low	Cross sectional	Laboratory	Active TB - culture confirmed	37	2010	12	Not reported	n/a
Luzzati 2011 [41]	Italy	Low	Retrospective cohort	Hospital	Pulmonary TB - sputum smear positive	112	2004–2008	60	not reported	11/13
Mansoori 2016 [42]	Iran	Medium	Cross sectional	Routinely collected health data	Active TB - culture confirmed new	176	2014–2015	13	not reported	9/13
Merza 2011 [43]	Iran	Medium	Retrospective cohort	Hospital	Active TB - culture confirmed with DST	1,742	2000–2005	55	not reported	11/13
Moosazadeh 2014 [44]	Iran	Medium	Cross sectional	Routinely collected health data	Active TB	73,945	2005–2011	84	not reported	8/13
Papakala 2017 [45]	Greece	Low	Retrospective cohort	Hospital	Active TB	88	2012–2014	36	not reported	9/13
Peghin 2017 [46]	Spain	Low	Retrospective cohort	Hospital	Extra-pulmonary TB - spinal	54	1993–2014	264	not reported	10/13
Ravan 2013 [47]	Iran	Medium	Cross sectional	TB clinic	Active TB	258	missing	–	not reported	4/13
Saavedra 2012 [48]	Spain	Low	Retrospective cohort	Hospital	Active TB	33	2010–2011	13	not reported	n/a
Sanghvi 2011 [49]	United Kingdom	Low	Retrospective cohort	Hospital	Extra-pulmonary TB - uveitis	19	1992–2007	184	not reported	10/13
Vanhomwegen 2011 [50]	United States	Low	Retrospective cohort	Community setting	Active TB - culture confirmed	109	1995–2004	120	not reported	10/13
Varghese 2013 [51]	Saudi Arabia	Low	Cross sectional	Laboratory	Active TB - culture confirmed	524	2009–2011	24	not reported	9/13

RoB = risk of bias

### 3.1 Quality assessment

Twenty-eight studies were assessed for quality and the remaining four studies could not be assessed as they were either an abstract or a surveillance report with no validated tool available. No study achieved a low risk of bias (RoB) score in all domains, with a median of 9 low risk domains out of a total of 13 domains assessed. (Table 1 and Table S4).

### 3.2 Outcomes

**Fig. 2:**
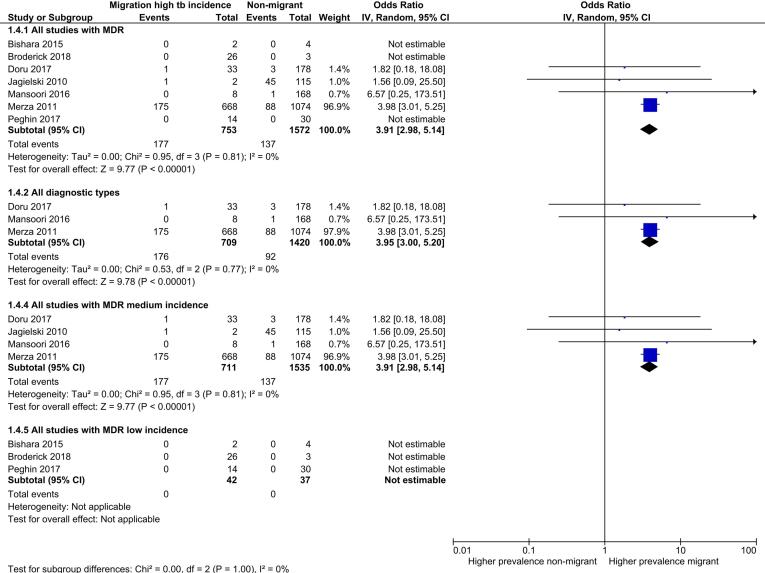
Meta-analysis of MDR-TB cases with subgroup and sensitivity analysis.

**Fig. 3:**
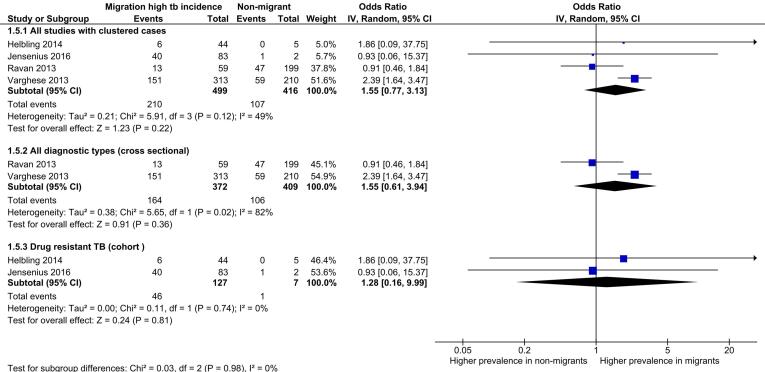
Meta-analysis of clustered TB cases with subgroup and sensitivity analysis.

**Fig. 4:**
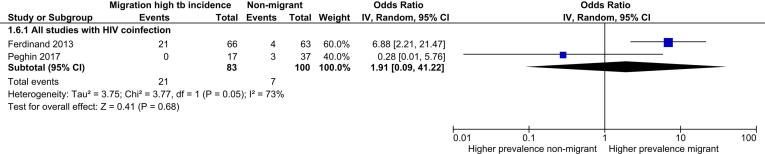
Meta-analysis of HIV coinfected TB cases.

**Fig. 5:**
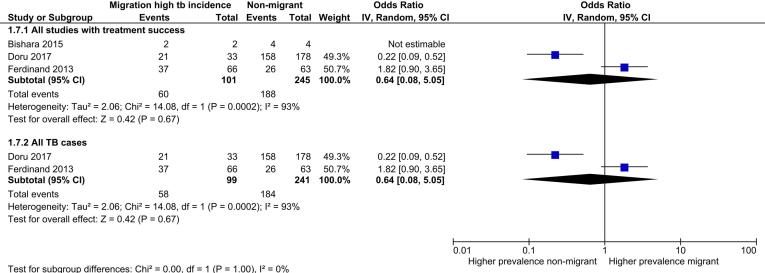
Meta-analysis of successfully treated TB cases with sensitivity analysis.

Seven review outcome measures were described in the 32 included studies with a total of 93,235 TB cases (median sample size = 98.5; range: 6–73,945). Ten studies reported only the primary review outcome and 22 studies described multiple secondary review outcome measures (Table S5). Of these 22 studies, six studies were not suitable for exploration via meta-analysis as the numerator was the same as the denominator. The remaining 16 studies reported between one and five secondary outcomes that were suitable to explore via meta-analysis. High levels of clinical, methodological and statistical heterogeneity were present among the included studies. Table 2 summarises the results of the analysis of secondary review outcomes while Fig. 2, Fig. 3, Fig. 4, Fig. 5 display the results of meta-analysis for secondary outcomes where differences in the proportions between high incidence migrants and non-migrants were detected. Meta-analysis results for secondary outcomes that did not have significant differences in the proportions between exposure groups are displayed as forest plots in Figures S2 and S3.

**Table 2:** Summary of secondary outcomes reported by included studies.

**Outcomes**	**Proportion high incidence migrants**	**Proportion non-migrants**	**Chi squared (P value)**	**Meta-analysis pooled odds ratio**	**Heterogeneity (I^2^)**	**Number of studies reporting outcome**
Sputum smear positive TB cases	0.71	0.70	0.010 (0.919)	1.17 (0.49–2.80)	0%	4
Case with any first line drug resistance	0.11	0.10	0.594 (0.441)	1.51 (0.81–2.82)	0%	8
MDR-TB cases	0.23	0.09	95.292 (<0.00001)	3.91 (2.98–5.14)	0%	7
Clustered cases	0.42	0.26	26.828 (<0.00001)	1.55 (0.77–3.13)	93%	3
HIV co-infected cases	0.19	0.05	14.393 (0.0001)	1.91 (0.09–41.22)	73%	5
Successfully treated cases	0.59	0.76	10.578 (0.001)	0.64 (0.08–5.05)	49%	3

In addition to data on high incidence migrants and non-migrants, 12 included studies also reported a total of 105 migrants from low to medium TB incidence countries. This corresponds to 0.1% of total active TB cases within the included studies and a range from 0.2 to 20.2%.

#### 3.2.1 Proportion high incidence migrant TB cases

The proportion of high incidence migrants among total active TB cases reported across studies ranged from 0.02 to 0.94. The overall proportion of high incidence migrants was 0.15 but the meta-analysis of this outcome produced a pooled proportion of 0.47. Subgroup and sensitivity analyses were unable to detect the source of the high levels of heterogeneity (I^2^ = 99%) present within the meta-analysis (Figure S1). Due to the uncertainty of these findings, they are provided for completeness and transparency within the supplementary materials but are not considered further within this article.

#### 3.2.2 Sputum smear positivity

Data on sputum smear positivity were reported by a total of five included studies. The overall proportion of sputum smear positivity was similar among high incidence migrants (0.71; range: 0.25–1.0) to non-migrants (0.70, range: 0.19–0.74). No difference was detected between the odds for this outcome and the statistical heterogeneity was low (I^2^ = 0%). Sensitivity analysis of studies that reported on all diagnostic types of TB found no significant difference between the two groups for this outcome (Figure S2) but the statistical heterogeneity increased to moderate (I^2^ = 49%).

#### 3.2.3 Resistance to any first line anti-TB drug

Data on drug resistance to any first line drug were reported by eight studies, six of which reported cases of drug resistance. No differences were detected in the overall proportions of high incidence migrants versus non-migrants (0.11 versus 0.10) among cases resistant to any first line anti-TB drug. The pooled odds ratio for this outcome was 1.51 [0.81–2.82] but no significant difference was detected between the two groups and the statistical heterogeneity was low (I^2^ = 0%). Sensitivity analysis of studies that reported on all diagnostic types of TB found no significant difference between the odds of high incidence migrants versus non-migrants (Figure S3) and the statistical heterogeneity remained similar (I^2^ = 0%).

#### 3.2.4 MDR-TB

Data on MDR-TB were reported by eight studies but only four studies reported MDR cases, all in medium incidence settings. The proportion of high incidence migrants with MDR-TB was significantly higher than the proportion of non-migrants with MDR in the four studies (0.23 versus 0.09, P: <0.001). This was reflected in the results of the meta-analysis with an increased odds of MDR among high incidence migrants compared to non-migrants (OR 3.91 [2.98–5.14]) with low levels of statistical heterogeneity found (I^2^ = 0%). The statistical heterogeneity remained similar when a sensitivity analysis restricting studies to the clinical category of all diagnostic types of TB and subgroup analysis by incidence level was performed (Fig. 2).

#### 3.2.5 Clustered cases

Data on clustered cases were reported by four included studies. Two studies were retrospective cohort studies looking at drug resistant TB cases while the remaining two studies were cross sectional studies which analysed all clinical presentations of TB. While the overall proportion of clustered cases was significantly higher among high incidence migrants compared to non-migrants (0.42 versus 0.26, P: <0.001), meta-analysis did not detect any significant difference between the odds ratios of exposure categories with moderate levels of heterogeneity detected (Fig. 3). The source of heterogeneity was explored further via subgroup and sensitivity analysis. Heterogeneity was reduced from 49% to 0% when one study was removed.[47] Removal of this study from the meta-analysis also resulted in significantly increased odds of the outcome in high incidence migrants. When subgroup analyses by study design was performed, heterogeneity was high for cross sectional study designs and low among cohort studies but the test for subgroup differences was not significant. It was not possible to stratify this subgroup analysis by the sensitivity analysis due to the small numbers of studies included. Neither sensitivity analysis (all diagnostic types and drug resistance only) detected any significant difference between the exposure categories but high levels of heterogeneity were present when only cross-sectional studies were combined.

#### 3.2.6 HIV coinfections

Data on HIV coinfection were reported by five included studies but only two of the studies had HIV coinfected cases. The overall proportion of HIV coinfection was significantly higher among high incidence migrants than non-migrants (0.19 versus 0.05, P: <0.001). Meta-analysis of HIV coinfection did not detect any significant difference between exposure categories (OR 1.91 [0.09–41.22]) and high levels of heterogeneity were present (Fig. 4). Results from the two included studies were also divergent in direction of effect. It was not possible to explore the source of this heterogeneity via subgroup analysis due to the low number of studies with HIV coinfected cases (n = 2) but clinical and methodological heterogeneity were observed in the clinical presentations, settings and geographical regions.

#### 3.2.7 Treatment success

Data on treatment success were reported by three included studies. Despite the overall proportion of treatment success being significantly lower among high incidence migrants than non-migrants (0.59 versus 0.76, p: <0.001) meta-analysis showed a high level of heterogeneity (I^2^ = 93%) and no significant difference between exposures or within subgroup analyses. Results from the two studies included in the meta-analysis were also divergent in direction of effect. (Fig. 5).

## 4 Discussion

While significant differences in overall proportions among high incidence migrants and non-migrants were observed in key epidemiological indicators; MDR-TB, HIV co-infected cases, clustered cases and successfully treated cases, the results of the meta-analyses only supported this finding for MDR-TB. These findings should be interpreted cautiously due to the high levels of clinical, methodological and statistical heterogeneity present among the included studies.

The high proportion (70%) of articles excluded due to not reporting data which would have allowed for stratification of migrant cases by TB incidence level illustrates a gap in the evidence base. The two most common approaches were to specify the most frequent countries of origin and report the often substantial remainder as “other” or else to report by geographical regions of origin. In the latter case, this usually resulted in use of regions that contained different TB incidence levels.

The overall proportion of high incidence migrants among the primary outcome of active TB cases, appears to have been influenced by the two largest studies which reported lower proportions of high incidence migrants.[28], [44] After excluding these studies, the proportion of high incidence migrants increased from 0.15 to 0.49, which is compatible with the pooled proportion of 0.47 produced by the meta-analysis. These figures are also similar to Pareek 2016 which reported a median proportion of 52% foreign-born TB notifications from all incidence levels for selected OECD countries.[3]

This review found a much lower proportion of low to medium incidence migrants (0.1%) among active TB cases than a previous large scale European study which found that other EU/EEA countries contributed to 2.4% of all migrant TB cases within the EU/ EEA, ranging from 0.05% in Bulgaria to 36.6% in Cyprus.[7] In this review, low to medium incidence migrants were mainly reported from US and Australian studies which may reflect their proximity to low and medium incidence migrant source countries.

The higher proportion of MDR-TB detected in high incidence migrants in this review mirrors results in the published literature but this difference in odd ratios disappears if Merza (Iran) is excluded as it has a high weighting due to its study size.[43] A recent systematic review of MDR-TB prevalence in Iran reported moderate levels (5% in new cases and 23% in retreatment cases) thought to be associated with migration from neighbouring high TB incidence countries, mainly from Afghanistan.[52], [53] It should be noted that while it is typical for most studies to report the proportion migrant MDR of the total MDR cases, this review has presented the proportion of high incidence migrant MDR of total high incidence migrant cases.

While equivalence was found between exposure groups for any resistance to first line anti-TB drugs, no data was extracted by this review on whether the cases were newly diagnosed or a mix of new and relapse. The unexpected divergence in the results for any first line resistance and MDR-TB found by this review are thought to be influenced by only two included studies reporting data suitable for inclusion in both meta-analyses.[29], [42]

The conflicting results for clustered cases appear to be impacted by the inclusion of a poor quality cross sectional study.[47] Removal of this study from the meta-analysis resulted in significantly increased odds of the outcome in high incidence migrants. All of the studies reporting clustered cases used molecular typing methods, which can overestimate the proportion of clustering in low incidence settings compared to WGS and often reflects lineages that are common in the country of origin but not in the host country.[59], [60], [61].

Large scale representative data on HIV co-infected TB cases remains elusive in many low TB incidence settings.[6] Although five studies reported data on HIV coinfection, only two studies reported cases of HIV coinfection with a single study reporting HIV coinfections in both high incidence migrants and non-migrants.[31] This study was set in Guadaloupe and may reflect the country’s proximity to migrant source countries with high TB and HIV prevalence, such as Haiti where the overall HIV adult prevalence rate was estimated at 1.9% in 2019.[54] Although the overall proportion of HIV coinfections were significantly different between the exposure groups, this finding was not supported by the meta-analysis results. The meta-analysis displays divergence in the direction of the odds ratios as Ferdinand reported 32% HIV coinfection among high incidence migrants while Peghin (Spain) reported 0%.

Although treatment success results were inconclusive, migrant status has previously been associated with unsuccessful treatment outcomes, particularly among subpopulations such as undocumented, homeless or incarcerated migrants.[55], [56] Potential explanations include returning to country of origin before treatment completion and language and/or economic barriers to health service access.[57], [58] Some high incidence countries also experience high levels of MDR, which reduce treatment success rates. One study within the treatment success meta-analysis reported levels of MDR that were almost double that of non-migrants (3% versus 1.7%) which may have affected the low proportion of treatment success among migrants.[29] However it is not clear why there is a lower rate of treatment success among non-migrants in the remaining study as no cases of MDR were reported by Ferdinand.

The similar proportions of sputum smear positivity (0.71 versus 0.70) found among the exposure groups may be part of the contributing factors leading to low rates of onward transmission documented from migrants to non-migrants. Previously published studies have found that migrant TB epidemiology continues to reflect the incidence rates in their country of origin.[3], [62], [63] This finding should also be taken in the context that certain high TB incidence migrant populations may have more paucibacillary disease due to higher HIV prevalence, increased extrapulmonary disease and higher paediatric rates compared to non-migrants in low incidence countries.[6], [64]

### 4.1 Strengths and limitations

The strengths of this review comprise the use of robust transparent methods including protocol publication, inclusion of different study designs and grey literature, along with the use of sensitivity and subgroup analysis to investigate sources of clinical and methodological heterogeneity. Multiple outcomes have been examined as key epidemiological indicators relating to potential for transmission (sputum smear positive cases, clustered cases and successfully treated cases) and clinical complexity (HIV co-infected and drug resistant cases). Although many conflicting recommendations exist with regard to systematic review and meta-analysis of observational studies, the applicable sections of the Cochrane Handbook, PRISMA and MOOSE reporting guidelines were followed.[12], [13], [65], [66]

The limitations of this review include the lack of denominators within included studies to calculate and compare incidence rates to assess true differences in exposure groups. A pragmatic approach was taken when choosing the time period in order to avoid cross over between incidence levels in the same country over different time periods, however this has necessarily limited the scope of the review. As no translation resources were available to the review team, 23 studies from eight languages were not reviewed at full text stage, over half of which were Spanish (Table S6). Due to the clinical heterogeneity of the included studies, it was not possible to stratify most of the meta-analysis of the secondary outcomes by subgroup analysis as this resulted in too few studies per subgroup for meaningful analysis. Similarly, the small number of studies within meta-analyses, prevented investigation of publication bias via funnels plots.

No randomised controlled trials (RCTs) were included in this review as the exposure of interest cannot be randomly assigned. Despite applying a robust quality appraisal process, none of the studies were found to be of high quality. Although many studies had an epidemiological focus, the majority reported the type of analysis performed rather than a defined study design. High risk of bias results were most commonly observed in domains assessing sample size justification, power calculation, confounding and blinding of outcome assessors to exposure status. With the exception of one study, data were retrospectively extracted, so data on outcomes and exposures were extracted at the same time.

## 5 Conclusions

This review has demonstrated that significant differences in key epidemiological indicators are present between high incidence migrants and non-migrants.

To our knowledge, this is the first systematic review to compare key epidemiological indicators between high incidence migrants and non-migrant TB cases. Previous studies have focused on migrant TB as a homogenous group, potentially missing key characteristics and failing to inform the tailored prevention and control response required to meet current TB elimination goals.[6], [11]

Despite an awareness that TB incidence levels within migrant source countries influence TB epidemiology within host countries, this study highlights that an important gap in the literature persists in the form of a lack of detailed data on migrant origin which is required to inform TB elimination programmes. The considerable heterogeneity present in the results indicates that a tailored, migrant inclusive approach should be taken when viewing the issue of TB prevention and control.

## CRediT authorship contribution statement

**Sarah Jackson:** Conceptualization, Investigation, Data curation, Formal analysis, Validation, Writing - original draft, Writing - review & editing. **Zubair Kabir:** Conceptualization, Data curation, Writing - review & editing. **Catherine Comiskey:** Conceptualization, Data curation, Writing - review & editing.

## Declaration of Competing Interest

The authors declare that they have no known competing financial interests or personal relationships that could have appeared to influence the work reported in this paper.
