# Supplementary material for: Effects of migration on tuberculosis epidemiological indicators in low and medium tuberculosis incidence countries: A systematic review
Source: J Clin Tuberc Other Mycobact Dis. 2021 Feb 22;23:100225. doi: 10.1016/j.jctube.2021.100225 (PMC7930366; doi:10.1016/j.jctube.2021.100225)
Supplement: Supplementary data 1 [file mmc1.docx]

# **Supplementary materials**

#### **Table S1: Inclusion and exclusion criteria**

| **Inclusion criteria** | **Exclusion criteria** |
| --- | --- |
| Human studies | Animal studies |
| Case control, cohort or cross-sectional studies | Case studies, case series |
| Cases diagnosed/ resident in countries with low/medium TB incidence (<21/100,000) | Cases diagnosed/ resident in countries with high TB incidence (>39/100,000) |
| Active TB | Latent TB |
| Migration from high TB incidence country (>39/100,000) to low or medium TB incidence country (<21/100,000) | Migration from rural to urban within countries |
| English language publications | Non-English language publications |
| Studies published after 2009 | Studies published prior to 2010 |
| Studies reporting country birth for >94% of foreign-born TB cases | Studies reporting country of birth for <95% of foreign-born TB cases |
| Studies reporting data on migrants and non-migrants | Studies reporting migrants only or non-migrant only |

#### **Table S2: Terms employed by the search strategy used in EMBASE (EBSCO)**

| **No.** | **Terms employed by the search strategy in EMBASE** |
| --- | --- |
| #9 | #8 AND [embase]/lim |
| #8 | #5 AND #6 AND #7 |
| #7 | #3 OR #4 |
| #6 | #1 OR #2 |
| #5 | active OR confirm* OR positiv* |
| #4 | migrant'/exp OR 'migrant' OR 'refugee'/exp OR 'refugee' OR  'immigration'/exp OR 'immigration' OR 'migration'/exp OR 'migration' OR 'population migration'/exp OR 'population migration' |
| #3 | transient*:ab,ti OR migrant*:ab,ti OR emigrant*:ab,ti OR immigrant*:ab,ti OR refugee*:ab,ti OR 'asylum seeker*':ab,ti OR emigration*:ab,ti OR migration*:ab,ti OR immigration*:ab,ti OR relocation*:ab,ti OR relocate*:ab,ti |
| #2 | 'tuberculosis'/exp OR 'tuberculosis' OR 'lupus vulgaris'/exp OR 'lupus vulgaris' OR 'pott s disease'/exp OR 'pott s disease' OR 'pott disease' |
| #1 | tuberculosis:ab,ti OR tuberculoses:ab,ti OR tuberculous:ab,ti OR tuberculoid:ab,ti OR 'koch s disease':ab,ti OR 'kochs disease':ab,ti OR 'koch disease':ab,ti OR 'potts disease':ab,ti OR 'pott s disease':ab,ti OR 'pott disease':ab,ti OR scrofula:ab,ti OR phthisis:ab,ti |

#### **Table S3: Variables used in data extraction tool**

| **Section** | **Variable name** | **Variable description** | **Data type** |
| --- | --- | --- | --- |
| **Study details** | Study ID | Generated by Covidence | Numeric |
|  | Reviewer details | CC or SJ or ZK | Drop down |
|  | Citation | Citation & DOI of report | Free text |
|  | Author contact | Corresponding author & institution | Free text |
|  | Author | First author | Free text |
|  | Year Report | Year study was published | Numeric |
|  | **Year Study*** | Year study was conducted | Numeric |
|  | **Study Setting*** | Description of setting in which study was conducted | Free text |
|  | **Study Design*** | Case control, cohort pro, cohort retro, cross-sectional, RCHD | Drop down |
|  | Study Duration Mo | Duration of study in months | Numeric |
|  | StudyFU Mo | Duration of follow up in months | Numeric |
|  | Funding | Source of funding for study | Free text |
|  | Study Aims | Description of aims and objectives of study | Free text |
| **Study population** | Inclusion/ exclusion criteria | Inclusion/ exclusion criteria of the study | Free text |
|  | Pop Age | Age range of study population or restrictions on age. | Free text |
|  | Pop Female | Number females reported in the study | Numeric |
|  | Pop Male | Number of males reported in study | Numeric |
|  | Pop Country | Name of country study conducted in, option for multiple | Drop down |
|  | **Pop Region*** | Euro/SSEAsia/E Asia/ Africa/LatAm/Oceania | Drop down |
|  | Pop Migrant source | Details of the main source countries of incoming migrants | Free text |
|  | Pop CoMorbid | Details of any co-morbidities in population | Free text |
|  | Pop SocioDemo | Details of any socio-demographics in population | Free text |
|  | Pop Ethnicity | Details of any ethnicities mentioned in population | Free text |
|  | **Pop TB incidence*** | TB incidence of country study conducted in (per 100,000 pop) | Numeric |
| **Primary outcome N** | Total Active TB cases | Total number of active TB cases reported by study |  |
|  | Total Active TB cases Migrant HI | Total number of active TB cases in high incidence migrants reported by study |  |
|  | Total Active TB cases Migrant LMI | Total number active TB cases in high incidence migrants reported by study |  |
|  | Total Active TB cases Indig | Total number of active TB cases in non-migrants reported by study |  |
| **Primary outcome rates** | Active TB incidence rate | Active TB incidence rate reported by study | Numeric |
|  | Active TB incidence rate Migrant HI | Active TB incidence rate in high incidence migrants reported by study | Numeric |
|  | Active TB incidence rate Migrant LMI | Active TB incidence rate in low/medium incidence migrants reported by study | Numeric |
|  | Active TB incidence rate Indig | Active TB incidence rate in in non-migrants reported by study | Numeric |
| **2˚ outcome: Sputum smear positive cases** | SSPos Total | Total number of sputum smear positive cases reported by study | Numeric |
|  | SSPos Mig HI | Total number of sputum smear positive cases in high incidence migrants reported by study | Numeric |
|  | SSPos Mig LMI | Total number of sputum smear positive cases in low/med incidence migrants reported by study | Numeric |
|  | SSPos Indig | Total number of sputum smear positive cases in non-migrants reported by study | Numeric |
| **2˚ outcome: Clustered cases** | Clustered Total | Total number of clustered cases reported by study | Numeric |
|  | Clustered Mig HI | Total number of clustered cases in high incidence migrants reported by study | Numeric |
|  | Clustered Mig LMI | Total number of clustered cases in low/med incidence migrants reported by study | Numeric |
|  | Clustered Indig | Total number of clustered cases in non-migrants reported by study | Numeric |
| **2˚ outcome: Successfully treated cases** | TxSuccess Total | Total number of successfully treated cases reported by study | Numeric |
|  | TxSuccess Mig HI | Total number of successfully treated cases in high incidence migrants reported by study | Numeric |
|  | TxSuccess Mig LMI | Total number of successfully treated cases in low/med incidence migrants reported by study | Numeric |
|  | TxSuccess Indig | Total number of successfully treated cases in non-migrants reported by study | Numeric |
| **2˚ outcome: HIV co-infections** | TB HIV total | Total number of TB HIV coinfections reported by study | Numeric |
|  | TB HIV total | Total number TB HIV coinfections in high incidence migrants reported by study | Numeric |
|  | TB HIV total | Total number of TB HIV coinfections in low/med incidence migrants reported by study | Numeric |
|  | TB HIV total | Total number of TB HIV coinfections in non-migrants reported by study | Numeric |
| **2˚ outcome: Cases resistant to any first to anti-TB drug** | AnyRes Total | Total number of MDR cases reported by study | Numeric |
|  | AnyRes Mig HI | Total number of MDR cases in high incidence migrants reported by study | Numeric |
|  | AnyRes Mig LMI | Total number of MDR cases in low/med incidence migrants reported by study | Numeric |
|  | AnyRes Indig | Total number of MDR cases in non-migrants reported by study | Numeric |
| **2˚ outcome: MDR cases** | MDR Total | Total number of MDR cases reported by study | Numeric |
|  | MDR Mig HI | Total number of MDR cases in high incidence migrants reported by study | Numeric |
|  | MDR Mig LMI | Total number of MDR cases in low/med incidence migrants reported by study | Numeric |
|  | MDR Indig | Total number of MDR cases in non-migrants reported by study | Numeric |
| **Comments** | Comments | General comments and relevant references identified from bibliography | Free text |

#### **Table S4: Quality appraisal results of included studies**

| **Author** | **1. Research question or objective clearly stated?** | **2. Study population clearly defined?** | **3. Participation rate of eligible persons >49%?** | **4. Subjects selected from similar populations? Inclusion/ exclusion criteria pre-specified and uniformly applied?** | **5. Sample size justification, power description, variance or effect estimate provided?** | **6 Was exposure of interest measured prior to the outcome(s) being measured** | **7. Was the timeframe sufficient for outcomes to occur?** | **8. Did the study measure different levels of exposure?** | **9. Were exposure measures clearly defined, valid, reliable and implemented consistently across participants?** | **10. Was the exposure assessed more than once over time?** | **11. Were outcome measures clearly defined, valid, reliable, and implemented consistently across participants?** | **12. Outcome assessors blinded to exposure status of participants?** | **13. Loss to follow up after baseline <21%** | **14. Key potential confounders measured and adjusted statistically for their impact on the relationship between exposure and outcome?** |
| --- | --- | --- | --- | --- | --- | --- | --- | --- | --- | --- | --- | --- | --- | --- |
| Aguayo | n/a | n/a | n/a | n/a | n/a | n/a | n/a | n/a | n/a | n/a | n/a | n/a | n/a | n/a |
| Al-Hajoj | low | low | unclear | low | high | high | high | low | low | n/a | low | high | unclear | high |
| Azarkar | low | low | unclear | low | high | high | low | low | low | n/a | low | unclear | unclear | high |
| Bartu | low | low | low | low | high | low | low | low | low | n/a | low | high | low | high |
| Bendayan | low | low | low | low | high | high | low | low | low | n/a | low | high | low | high |
| Bishara | low | low | low | low | high | high | low | low | low | n/a | low | high | low | high |
| Broderick | low | low | low | low | high | high | low | low | low | n/a | low | high | low | high |
| Coll | low | low | low | low | high | low | low | low | unclear | n/a | low | high | low | high |
| Cruz-Ferro | low | low | low | low | high | unclear | low | low | high | n/a | low | high | low | high |
| Doĝru | low | low | low | low | high | low | low | low | low | n/a | low | high | low | high |
| Fallico | low | low | low | low | high | high | low | low | low | n/a | low | high | low | high |
| Ferdinand | low | low | low | low | high | low | low | low | low | n/a | low | high | low | high |
| Goblirsch | low | low | low | low | high | high | low | low | low | n/a | low | high | low | high |
| Helbling | low | low | low | low | high | low | low | low | low | n/a | low | high | low | high |
| Jagielski | low | low | low | low | high | low | low | low | low | n/a | low | high | low | high |
| Jawad | low | low | low | low | high | high | low | low | low | n/a | low | high | low | high |
| Jensenius | low | low | low | low | high | low | low | low | low | n/a | low | high | low | high |
| Jones | n/a | n/a | n/a | n/a | n/a | n/a | n/a | n/a | n/a | n/a | n/a | n/a | n/a | n/a |
| Kentley | low | low | low | low | high | low | low | unclear | low | n/a | unclear | high | low | high |
| Krogh | low | low | low | low | high | low | low | low | low | n/a | low | high | low | high |
| Lumb | n/a | n/a | n/a | n/a | n/a | n/a | n/a | n/a | n/a | n/a | n/a | n/a | n/a | n/a |
| Luzzati | low | low | low | low | high | low | low | low | low | n/a | low | high | low | low |
| Mansoori | low | low | low | low | high | low | low | low | low | n/a | low | high | low | high |
| Merza | low | low | low | low | high | low | low | low | low | n/a | low | high | low | low |
| Moosazadeh | low | low | low | low | high | high | low | low | low | n/a | low | high | unclear | high |
| Papakala | low | low | low | low | high | low | low | low | low | n/a | low | high | high | high |
| Peghin | low | low | low | low | high | low | low | low | low | n/a | low | high | low | high |
| Ravan | low | unclear | unclear | low | high | high | high | low | low | n/a | unclear | high | unclear | high |
| Saavedra | n/a | n/a | n/a | n/a | n/a | n/a | n/a | n/a | n/a | n/a | n/a | n/a | n/a | n/a |
| Sanghvi | low | low | low | low | high | low | low | low | low | n/a | low | high | low | high |
| Vanhomwege | low | low | low | low | high | low | low | low | low | n/a | low | high | high | low |
| Varghese | low | low | low | low | high | high | low | low | low | n/a | low | high | low | high |

#### **Table S5: Outcomes reported by included studies**

| **Study name** | **Clinical group** | **Primary outcome** | | | | **Secondary outcomes for consideration via meta-analysis^[[1]](#footnote-1)^** |
| --- | --- | --- | --- | --- | --- | --- |
|  |  | **Total active TB cases** | **Migrants - HI^[[2]](#footnote-2)^  n (%)** | **Migrants - LMI  n (%)** | **Non-migrants n (%)** |  |
| Aguayo | Extrapulmonary TB | 20 | 15 (75) | 0 (0) | 5 (25) | None |
| Al-Hajoj | Extrapulmonary TB | 381 | 135 (35) | 22 (5) | 224 (58) | None |
| Azarkar | Pulmonary TB | 85 | 6 (7) | 0 (0) | 79 (92) | None |
| Bartu | Drug resistant | 50 | 16 (32) | 1 (2) | 33 (66) | HIV pos |
| Bendayan | Drug resistant | 132 | 120 (90) | 7 (5) | 5 (3) | None |
| Bishara | Pregnancy TB | 6 | 2 (33) | 0 (0) | 4 (66) | SS pos, HIV pos, Any Res, MDR, Tx Success |
| Broderick | Extrapulmonary TB | 29 | 26 (89) | (0) | 3 (10) | Any res, MDR |
| Coll | Donors & recipients | 6 | 1 (16) | 0 (0) | 5 (83) | None |
| Cruz-Ferro | All diagnostic types | 12,615 | 287 (2) | 0 (0) | 12,328 (97) | None |
| Dogru | All diagnostic types | 211 | 33 (15) | 0 (0) | 178 (84) | SS pos, Any Res, MDR, Tx Success |
| Fallico | Clustered cases | 339 | 175 (51) | 18 (5) | 146 (43) | None |
| Ferdinand | All diagnostic types | 129 | 66 (51) | 0 (0) | 63 (48) | SS pos, HIV pos, Any Res, Tx Success |
| Goblirsch | Extrapulmonary TB | 39 | 27 (69) | 0 (0) | 12 (30) | None |
| Helbling | Drug resistant | 51 | 44 (86) | 2 (3) | 5 (9) | Clustered |
| Jagielski | Drug resistant | 117 | 2 (1) | 0 (0) | 115 (98) | MDR |
| Jawad | All diagnostic types | 1584 | 1138 (71) | (0) | 446 (28) | None |
| Jensenius | Drug resistant | 88 | 83 (94) | 3 (3) | 2 (2) | Clustered |
| Jones | All diagnostic types | 171 | 127 (74) | 17 (9) | 27 (15) | None |
| Kentley | Extrapulmonary TB | 61 | 55 (90) | 2 (3) | 4 (6) | None |
| Krogh | Paediatric TB | 24 | 8 (33) | 0 (0) | 16 (66) | SS pos, HIV pos, Any Res, MDR |
| Lumb | Drug resistant | 37 | 34 (91) | 0 (0) | 3 (8) | None |
| Luzzati | Pulmonary TB | 112 | 56 (50) | 0 (0) | 56 (50) | None |
| Mansoori | All diagnostic types | 176 | 8 (4) | 0 (0) | 168 (95) | Any res, MDR |
| Merza | All diagnostic types | 1742 | 668 (38) | 0 (0) | 1074 (61) | MDR |
| Moosazadeh | All diagnostic types | 73,945 | 10377 (14) | 0 | 63,568 (85) | None |
| Papakala | All diagnostic types | 88 | 18 (20) | 9 (10) | 61 (69) | Any Res |
| Peghin | Extrapulmonary TB | 54 | 17 (31) | 0 (0) | 37 (68) | HIV pos, Any Res, MDR |
| Ravan | All diagnostic types | 258 | 59 (22) | 0 (0) | 199 (77) | Clustered |
| Saavedra | All diagnostic types | 33 | 13 (39) | 1 (3) | 19 (57) | None |
| Sanghvi | Extrapulmonary TB | 19 | 16 (84) | 0 (0) | 3 (15) | SS pos |
| Vanhomwegen | Clustered cases | 109 | 35 (32) | 22 (20) | 52 (47) | None |
| Varghese | All diagnostic types | 524 | 313 (59) | 1 (0) | 210 (40) | Clustered |

#### **Table S6: List of studies excluded on basis of language restrictions**

1. [Tuberculosis Annual Report 2012. (1). Summary of tuberculosis notification statistics and foreign-born tuberculosis patients]. Kekkaku: [Tuberculosis]. 2014;89(6):619-25.

2. Ayerra MG, Gorostidi AM, Aguirre MH, Setas AG. Tuberculous disease in pediatrics: Ten years’ experience. Pediatria de Atencion Primaria. 2019;21(82):e61-e6.

3. Berzosa Sánchez A, Illán Ramos M, Prados M, Calderón Gallego C, Francisco González L, Callejas Caballero I, et al. Tuberculosis in the paediatric population of Madrid in the last 26 years. Enfermedades Infecciosas Y Microbiologia Clinica. 2019.

4. Chalela R, Sánchez-Font A, Domínguez-Álvarez M, Badenes-Bonet D, Pijuan L, Curull V. [Role of endobronchial ultrasound-guided transbronchial needle aspiration in the diagnosis of mediastinal tuberculosis]. Medicina Clinica. 2016;146(12):532-5.

5. Dahle UR, Sandven P, Heldal E, Caugant DA. [Genetic analysis of Mycobacterium tuberculosis in Norway 1994-98]. Tidsskrift For Den Norske Laegeforening: Tidsskrift For Praktisk Medicin, Ny Raekke. 2002;122(7):697-700.

6. Fica A, Herrera T, Aguilera X. [Worsening situation of tuberculosis in Chile]. Revista Medica De Chile. 2019;147(8):1042-52.

7. Fourkas D, Deliolanis I, Pantazatou A, Smilakou S, Bekris E, Spalla G, et al. Insufficiency in the reference and surveillance procedure of tuberculosis in a tertiary hospital. Acta Microbiologica Hellenica. 2012;57(3-4):35-42.

8. García Ayerra M, Mosquera Gorostidi A, Herranz Aguirre M, Gil Setas A. Enfermedad tuberculosa en la edad pediátrica: experiencia de diez años. Pediatría Atención Primaria. 2019;21(82):e61-e6.

9. García Sánchez I, Pérez de Oteyza C, Gilsanz Fernández C. [Tuberculosis epidemiological study in a third level hospital during 2001]. Anales De Medicina Interna (Madrid, Spain: 1984). 2005;22(5):222-6.

10. García-Zamalloa AM, Arrizabalaga J. [Tuberculosis in the Bajo Deba area (Guipúzcoa, Spain) from 1995 to 2006]. Enfermedades Infecciosas Y Microbiologia Clinica. 2008;26(4):187-93.

11. Gil M, Moreno R, Marín M, Romeu MÁ, Gomila B, González F. [Influence of immigration on tuberculosis transmission patterns in Castellón, Spain (2004-2007)]. Gaceta Sanitaria. 2011;25(2):122-6.

12. Ikeda K, Nishikiori H, Kondo S, Kobayashi T, Taya T, Mori Y, et al. [THE CURRENT SITUATION OF FOREIGN TUBERCULOSIS PATIENTS AND THEIR CONCURRENT HIV INFECTION IN HOKKAIDO]. Kekkaku: [Tuberculosis]. 2016;91(2):33-9.

13. Kubín M, Malý M, Jágrová Z. Contribution of homeless people to the incidence of tuberculosis in Prague. Studia Pneumologica et Phthiseologica. 2016;76(2):67-72.

14. Margarit A, Simó S, Rozas L, Deyà-Martínez À, Barrabeig I, Gené A, et al. [Adolescent tuberculosis; a challenge and opportunity to prevent community transmission]. Anales De Pediatria (Barcelona, Spain: 2003). 2017;86(3):110-4.

15. Masvidal Aliberch RM, Gil BM, Vall Mayans M, Zabaleta Del Olmo E, Carnero Olmedo E, Rodríguez De La Rubia Rodríguez-Manzaneque CR. Tuberculous infection from an area of high incidence of tuberculosis and with a high proportion of immigrants. Anales de Pediatria. 2004;60(1):22-7.

16. Morales-García C, Parra-Ruiz J, Valero-Aguilera B, Sanbonmatsu-Gámez S, Sánchez-Martínez JA, Hernández-Quero J. [Characteristics of tuberculosis in the immigrant population in South Granada Health Area]. Enfermedades Infecciosas Y Microbiologia Clinica. 2015;33(3):166-72.

17. Morgado A, Köhnenkampf R, Navarrete P, García P, Balcells ME. [Clinical and epidemiological profile of tuberculosis in a university hospital in Santiago, Chile]. Revista Medica De Chile. 2012;140(7):853-8.

18. Pape S, Groß F, Ulrichs T. [The tuberculosis situation in the Berlin prison system from 2011-2016-a follow-up study]. Bundesgesundheitsblatt, Gesundheitsforschung, Gesundheitsschutz. 2019;62(7):893-903.

19. Peï¿½a M C, Caamaï¿½o M R, Mesa M MJ, Urzï¿½a S R, Pinochet R M, Miranda M C. Contribution of immigrants to tuberculosis in a health service in Chile. Revista Chilena de Enfermedades Respiratorias. 2016;32(1):41-9.

20. Priwitzer M. Tuberculosis in Germany: Epidemiology, old and new drawbacks and potential solutions. Atemwegs- und Lungenkrankheiten. 2016;42(3):105-14.

21. Rodrigo T, García-García J-M, Caminero JA, Ruiz-Manzano J, Anibarro L, García-Clemente MM, et al. Evaluation of the Integrated Tuberculosis Research Program Sponsored by the Spanish Society of Pulmonology and Thoracic Surgery: 11 Years on. Archivos De Bronconeumologia. 2019.

22. Ruffini E, Compagnoni L, Tubaldi L, Infriccioli G, Vianelli P, Genga R, et al. [Congenital and perinatal infections in the Marche region (Italy): an epidemiological study and differences between ethnic groups]. Le Infezioni In Medicina: Rivista Periodica Di Eziologia, Epidemiologia, Diagnostica, Clinica E Terapia Delle Patologie Infettive. 2014;22(3):213-21.

23. Saghafipour A, Noroozei M, Mostafavi R, Heidarpour A, Ghorbani M. The epidemiologic status of pulmonary tuberculosis and its associated risk factors in qom province during 2002-2010. Journal of Mazandaran University of Medical Sciences. 2012;22(90):63-70.

#### **Figure S1: Proportion of active TB cases in high incidence migrants of total study population with sensitivity analysis by clinical category^[[3]](#footnote-3)^**


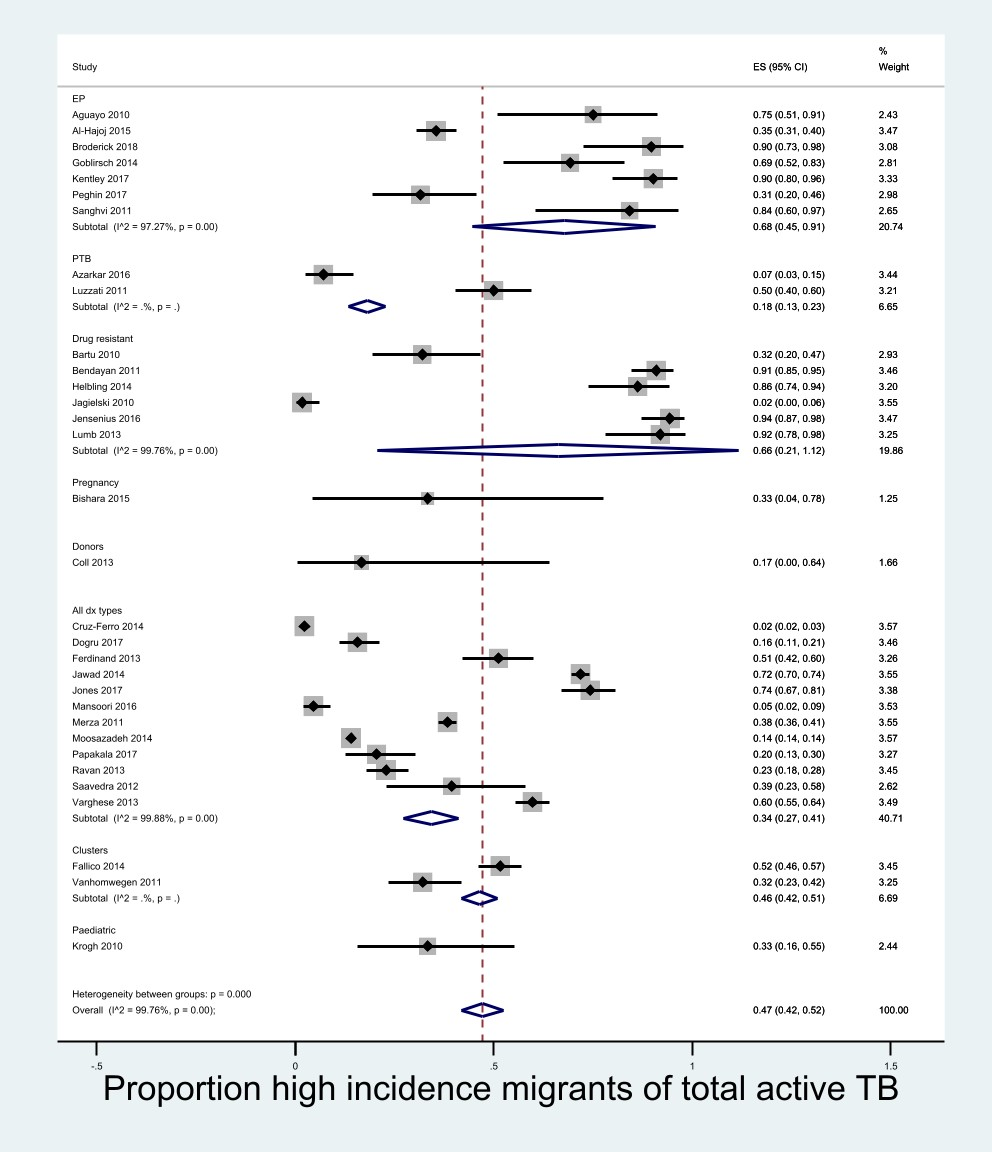


#### **Figure S2: Meta-analysis of sputum smear positive TB cases with sensitivity analysis**


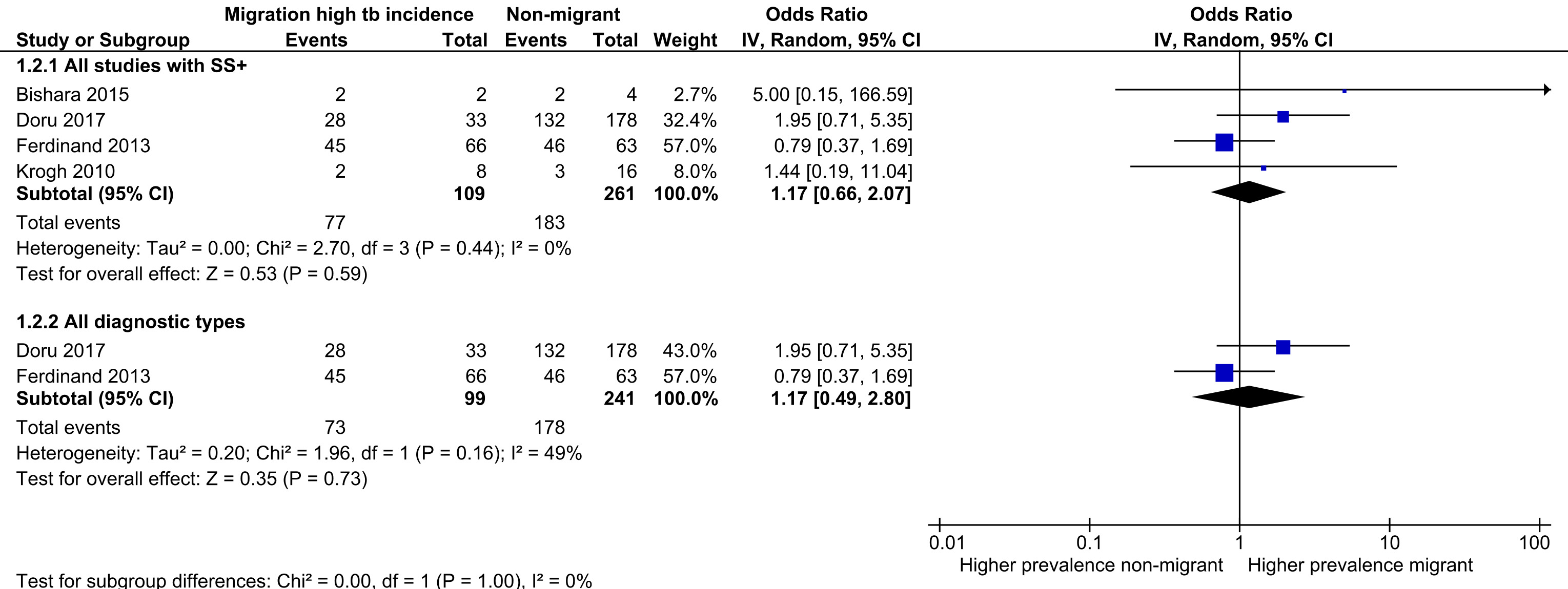


#### **Figure S3: Meta-analysis of TB cases with first line anti-TB drug resistance with sensitivity analysis**


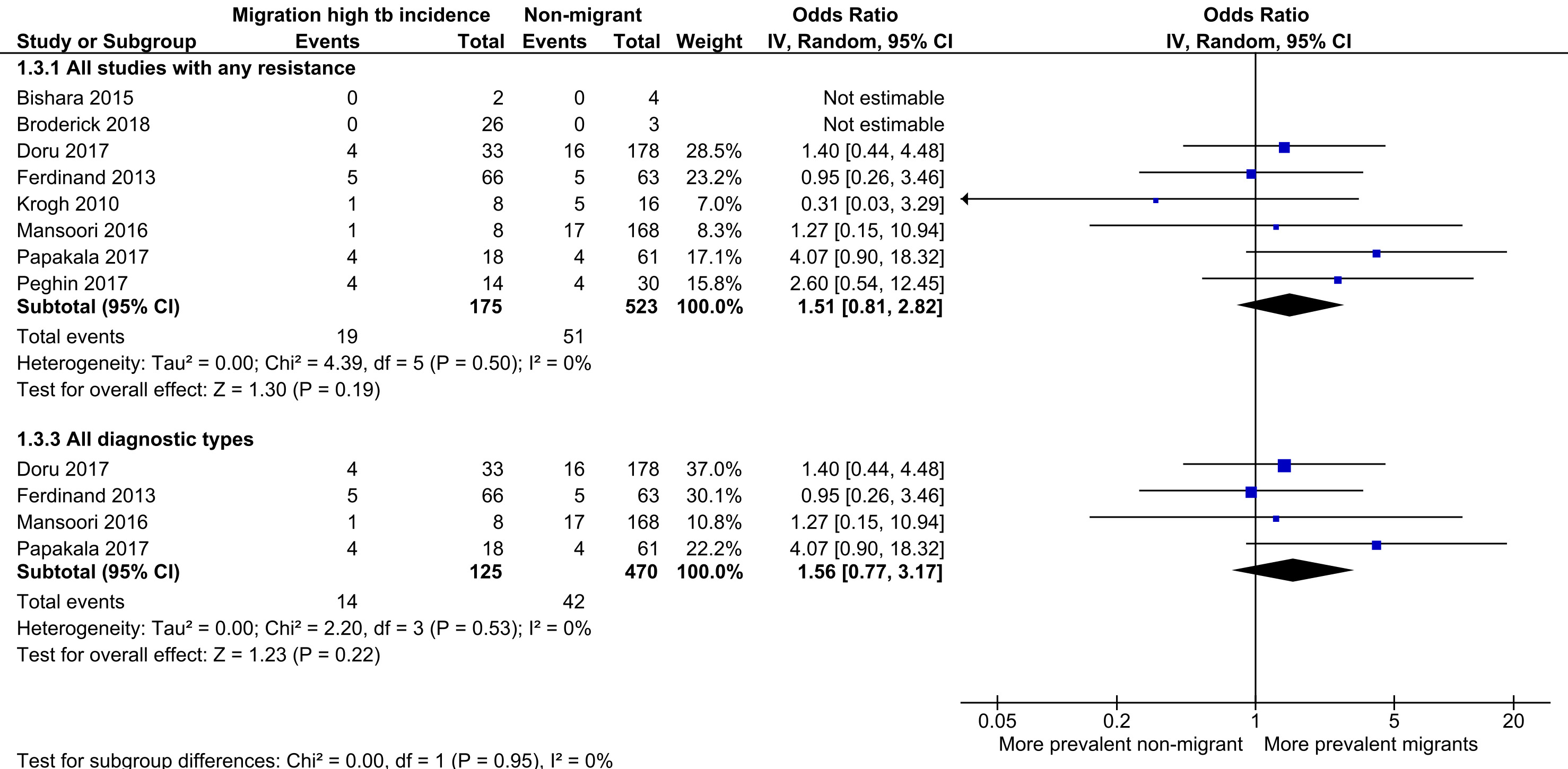


1. Where Micro conf = microbiologically confirmed cases; SS Pos = sputum smear positive cases; HIV pos = HIV co-infected cases; Any Res = cases with resistance to any 1^st^ line anti-TB drug; MDR – multi-drug resistant TB; Clustered = cases clustered by molecular or whole genome sequencing; Tx Success = successfully treated cases. [↑](#footnote-ref-1)
2. Where HI = high TB incidence migrants; LMI = low to medium TB incidence migrants [↑](#footnote-ref-2)
3. Where EP = extrapulmonary, PTB = pulmonary TB and all dx types = all diagnostic types [↑](#footnote-ref-3)
